# Thymidine exerts anti-doxorubicin-induced cardiomyopathy effect through the regulation of the PPAR signaling pathways and ferroptosis pathways

**DOI:** 10.3389/fphar.2025.1524167

**Published:** 2025-09-30

**Authors:** Bin Li, Qifei Wang, Jiashuo Zhou, Peihai Li, Chen Sun, Qing Xia, Yun Zhang

**Affiliations:** ^1^ Biology Institute, Qilu University of Technology (Shandong Academy of Sciences), Jinan, China; ^2^ Shandong Zebrafish Human Disease Model and Drug Screening Engineering Technology Research Center, Jinan, China

**Keywords:** thymidine, doxorubicin, cardiomyopathy, zebrafish, PPAR signaling pathway, ferroptosis pathway

## Abstract

**Introduction:**

To evaluate the anti-cardiomyopathic activity of thymine (Thy) and to elucidate its mechanism of action.

**Methods:**

Transgenic zebrafish with enhanced green fluorescent protein (EGFP)-labelled hearts (*Tg (cmlc2: EGFP)*) and wild-type AB zebrafish were used as experimental animals. A blank control group, a doxorubicin (DOX) model group, a dexrazoxane (DEX)-positive drug group and Thy drug treatment group were established. After treatment, indicators closely related to cardiac function, such as the pericardial area, heart rate, stroke volume, short-axis shortening (SAS) rate, and ejection fraction of the zebrafish in each group, were evaluated to determine the protective activity of Thy against DOX-induced cardiomyopathy. The regulatory roles of key genes in the pathways associated with the cardioprotective activity of Thy were analyzed via RT-qPCR.

**Results:**

The results indicated that Thy effectively relieved DOX-induced pericardial edema; reversed the effects of DOX on heart rate, stroke volume, SAS rate, ejection fraction, and blood flow velocity; and relieved DOX-induced myocardial ischemia, myocardial cell apoptosis and pathological structural changes in heart tissues. The RT‒qPCR results revealed that Thy regulated the mRNA expression levels of genes related to the PPAR signaling pathway and ferroptosis pathway (such as *pparg, apoa1a, acsl5, pltp,* and *tfa*).

**Discussion:**

Thy may exert its anti-DOX-induced cardiomyopathy effect through the regulation of the PPAR signaling and ferroptosis pathways.

## Graphical Abstract

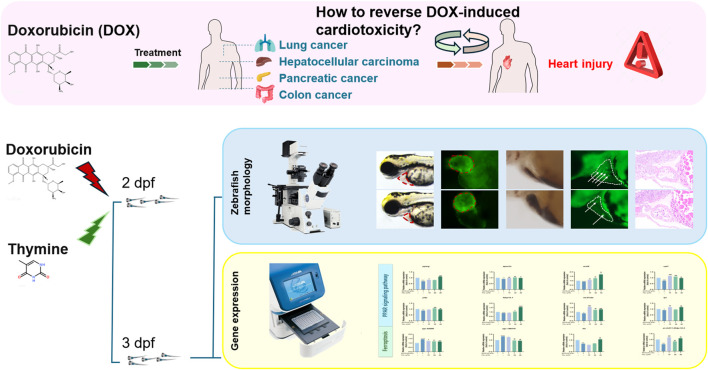

## Introduction

Doxorubicin (DOX) is a cancer chemotherapeutic drug with a broad antitumor spectrum, but DOX treatment can cause adverse reactions. DOX can cause dilated cardiomyopathy (DCM), greatly limiting its clinical use (Bi et al., 2022; Ding et al., 2023). The main clinical features of DCM are ventricular dilatation, systolic dysfunction, arrhythmias, myocardial ischemia and myocardial cell apoptosis. In patients with cardiomyopathy, the ventricular wall gradually becomes thinner, and symptoms such as arrhythmias and atrioventricular block disorder can be detected in patients via ultrasound and other techniques. Dexrazoxane (DEX) is currently the only drug approved by the Food and Drug Administration (FDA) to reduce DOX-induced cardiomyopathy. However, DEX has a short half-life in plasma and has adverse effects, such as allergies and liver damage, limiting its clinical application (Chen et al., 2022; Maciag et al., 2022; Cheng et al., 2022). Therefore, innovative drugs for the treatment of DOX-induced cardiomyopathy are urgently needed.

The zebrafish and human genomes are highly homologous, and the structure and function of the heart are highly similar between zebrafish and mammals. The heart is composed of atria and ventricles, and its relaxation and contraction mechanisms are highly conserved (Rosa et al., 2022). Compared with that of mice, the heart rate (120–140 beats/min) of zebrafish is closer to that of humans (Santiago et al., 2021). Because the body of zebrafish larvae is transparent, the beating of the heart and blood flow can be directly observed in enhanced green fluorescent protein (EGFP)-labeled transgenic zebrafish through a microscope, allowing the calculation of the heart rate, pericardial area, stroke volume, and short-axis shortening. Ejection fraction, blood flow velocity and other indicators are used to rapidly evaluate cardiac function. Moreover, the reaction of zebrafish toward cardiotoxic drugs is similar to that of humans. In zebrafish, cardiotoxic drugs can cause cardiac dysfunction and morphological changes in heart tissue, and zebrafish can survive for a period of time with heart damage. These advantages make zebrafish a superior model for studying cardiac function (Zakaria et al., 2018; Huang et al., 2020).

Thymine (Thy) is a pyrimidine base isolated from the thymus and is the raw material of trifluorothymidine, an antinucleic acid metabolism antitumor drug (Su et al., 2023). The cardioprotective activity and anticardiomyopathy activities of Thy have not been reported. In this study, DOX was used to establish a zebrafish cardiomyopathy model to evaluate the protective activity of Thy against DOX-induced heart injury and to elucidate its mechanism of action, thus providing a reference for the prevention and treatment of DOX-induced cardiomyopathy.

## Materials and methods

### Chemicals

DOX (CAS number: 23214-92-8, lot number: A396641337769) was purchased from APE × BIO (United States). DEX (CAS number: 24584-09-6, lot number: 0448942-18) was purchased from Cayman (United States). Thy (CAS number: 65-71-4, lot number: C14851700) was purchased from Shanghai Macklin Biochemical Co., Ltd.

### Zebrafish husbandry

*Tg (cmlc2: EGFP)* and wild-type AB zebrafish with EGFP-labeled hearts were provided by the Drug Screening Platform of the Institute of Biology, Shandong Academy of Sciences. Male and female zebrafish were housed separately at 28 °C under standard conditions and a 14 h light/10 h dark cycle. After natural mating, embryos were collected, transferred to zebrafish culture water (5 mM NaCl, 0.17 mM KCl, 0.33 mM CaCl_2_, and 0.33 mM MgSO_4_) containing 0.02% methylene blue, and placed in a constant-temperature light incubator (SPX-280B-G, Shanghai Genestar Biotechnology Co., Ltd., Shanghai, China) at 28.0 °C ± 0.5 °C. The culture water was changed every 24 h. All experiments were performed in accordance with standard ethical guidelines and approved by the Animal Ethics Committee of the Institute of Biology, Qilu University of Technology, under animal welfare ethics review approval number SWS20220929.

### Drug grouping

*Tg (cmlc2: EGFP)* zebrafish that had developed normally at 48 hpf were selected and placed in 24-well plates, with 15 juveniles per well. In the pilot experiment, a blank control group (zebrafish culture water), a DOX model group (60 μM DOX), a DEX-positive control group (60 μM DOX + 10 μM DEX) and Thy-treated group (60 μM DOX + 10, 20 or 40 μM Thy) were used. The wells for each group were set up in triplicate, and the plates were placed in a light incubator for 24 h.

### Observation of the morphology of zebrafish hearts

Changes in the size of the pericardial area directly reflect abnormalities in the zebrafish heart. Opening of the S-loop of the heart results in a greater sinus venous–glomus arteriae (SV-BA) distance. Therefore, the pericardial area and SV-BA distance, as measured under a microscope, can serve as direct indicators of zebrafish heart abnormalities (Wu et al., 2023; Li et al., 2021). Twenty-four hours after drug administration, the zebrafish were anesthetized with an anesthetic agent (0.3% tricaine by mass concentration) and then fixed with 4% methylcellulose. After anesthesia, pictures were taken from the side under a fluorescence stereo microscope (Olympus, SZX2-ILLTQ, Tokyo, Japan), and the morphology of the hearts of zebrafish in each group was photographed and recorded. The SV-BA distance and pericardial area were measured using Image-Pro Plus 6.0 software.

### Observation of transgenic zebrafish with green fluorescence-labeled hearts

The clinical manifestations of DCM are arrhythmia, ventricular dilatation, and systolic dysfunction. Heart rate, stroke volume, short-axis shortening rate, and ejection fraction can reflect heart rhythm, ventricular dilatation, and systolic function (Begay et al., 2016). Twenty-four hours after drug administration, the zebrafish were fixed in 4% methylcellulose. After fixation, a video of the heartbeat was recorded under an inverted fluorescence microscope (Olympus, SZX16, Tokyo, Japan) with zebrafish in a prone position, and images of ventricular end-diastole and end-systole were extracted. The lengths of the major and minor axes at end-diastole and end-systole were measured using Image-Pro Plus 6.0 software, and the stroke volume, SAS rate and ejection fraction were calculated (Song et al., 2020).

### Measurement of blood flow velocity in zebrafish

Patients with cardiomyopathy often experience heart failure, resulting in cardiac pump dysfunction. In this study, blood flow velocity was measured to evaluate the effect of Thy on the blood flow velocity of zebrafish with DOX-induced heart injury (Eriksson et al., 2013). Twenty-four hours after drug administration, the zebrafish were anesthetized and fixed with 4% methylcellulose. Arterial blood flow velocity was measured using a blood flowmeter. Video of blood flow was analyzed, and the blood flow velocity was calculated using MicroZebraLab BloodFlow 3.4.6 software (ViewPoint, Lyon, France) (Gao et al., 2023).

### O-dianisidine erythrocyte staining

O-dianisidine binds to hemoglobin and forms a rust red precipitate in cells. In this study, O-dianisidine was used to analyze hemoglobin production in zebrafish. Twenty-four hours after drug administration, the zebrafish were stained with 1 mg/mL O-dianisidine staining solution for 20 min, and 10 zebrafish were randomly selected from each group and observed under a fluorescence stereo microscope (Zeiss,AXIO-V16,Germany). The erythrocytes in the heart region were photographed. Image-Pro Plus 6.0 image processing software was used to analyze the area of erythrocyte staining in the heart quantitatively to evaluate the antimyocardial ischemic action of the studied compounds (Fan et al., 2022).

### Staining of apoptotic cells in zebrafish hearts (acridine orange (AO) staining/TUNEL staining)

For AO staining, 24 h after drug administration, prepared AO stock solution was added to each well containing zebrafish to reach a final concentration of 5 μg/mL, followed by incubation in an incubator at 28 °C for 30 min. The zebrafish were washed 3 times with phosphate buffered saline (PBS) and observed under a fluorescence stereo microscope to count the number of apoptotic cells in the heart.

For TUNEL staining, 24 h after drug administration, zebrafish were fixed in 4% paraformaldehyde and placed in a refrigerator at 4 °C overnight, after which 0.3% Triton X-100 and sodium citrate antigen retrieval solution (1X) were added at a ratio of 1:300 and subsequent incubation in a refrigerator at 4 °C for 10 min. Tdt enzyme and fluorescence labeling solution were mixed together at a ratio of 1:10. Fluorescence labeling mixture (50 μL) was added to each group of zebrafish, followed by incubation in the dark for 1.5 h in a 37 °C incubator. The zebrafish were observed under a fluorescence stereo microscope (Zeiss, AXIO-V16, Germany) to count the number of apoptotic cells in the heart.

### Hematoxylin and eosin (H&E) staining

In patients with cardiomyopathy, the ventricular wall gradually becomes thinner, and myocardial cells are disordered. In this study, H&E staining was used to examine whether the heart tissue of zebrafish exhibited pathological changes. Twenty-four hours after drug administration, 10 zebrafish were randomly selected from blank control group, DOX modeling group, and drug-treated group (60 μM DOX + 20 μM Thy) and fixed with 4% paraformaldehyde fixative solution. The zebrafish were subsequently dehydrated with an ethanol gradient and then cleared with xylene. The zebrafish were then embedded in paraffin and sectioned. The sections were deparaffinized in xylene, absolute ethanol, and alcohol in water successively and then stained with hematoxylin and eosin. After dehydration and mounting, the sections were observed under a fluorescence stereo microscope (Olympus, SZX2-ILLTQ, Tokyo, Japan), and heart tissue was photographed and analyzed.

### Oil red O staining

Twenty-four hours after drug administration, 10 zebrafish were randomly selected from blank control group, DOX model group (60 Μm DOX), and drug-treated groups (60 μM DOX + 10, 20 or 40 μM Thy) and were fixed with 4% paraformaldehyde. The next day, the zebrafish were removed from the fixative solution and dehydrated in propylene glycol. Freshly prepared 0.3% Oil red O solution was added to the zebrafish, followed by incubation for 6 h in the dark. Excess staining solution was removed by washing with propylene glycol and PBST. Lipid accumulation in the hearts of zebrafish was observed under a fluorescence stereo microscope (Olympus, SZX2-ILLTQ, Tokyo, Japan) and photographed (Xu et al., 2023).

### RT‒qPCR detection of changes in the expression levels of relevant genes

Twenty-four hours after treatment, 30 zebrafish from blank control group, DOX modeling group and drug-treated group (60 μM DOX + 20 μM Thy) were randomly selected and washed three times with enzyme-free water. The zebrafish were homogenized, and RNA was extracted using a FastPure Cell/Tissue Total RNA isolation kit V2 (Vazyme, Nanjing, China). cDNA was obtained via reverse transcription, and RT‒PCR was performed using ChamQ Universal SYBR qPCR master mix. The RT‒PCR amplification conditions were as follows: predenaturation at 95 °C for 30 s; denaturation at 95 °C for 10 s and annealing at 60 °C for 10 s, for a total of 40 cycles; and 95 °C for 15 s, 60 °C for 60 s, and 95 °C for 15 s, for 1 cycle. Relative quantitative analysis was performed using *β-actin* as an internal reference gene. The PCR primers for the internal reference gene *β-actin* and the target genes were synthesized and purified by BioSune Biotechnology Co., Ltd. (Shanghai, China) and passed quality inspection. Primers for all genes are listed in Supplementary Table S1.

### Data analysis

The experimental data are expressed as means ± SD, and significant differences between groups were determined using t tests. *p* < 0.05 indicated a significant difference, and *p* < 0.01 indicated a highly significant difference. Statistical analyses were performed using GraphPad Prism 8.0 and Image-Pro Plus 6.0 software.

## Results

### Effects of thy on the morphology of the hearts of zebrafish with DOX-induced cardiomyopathy

**FIGURE 1:**
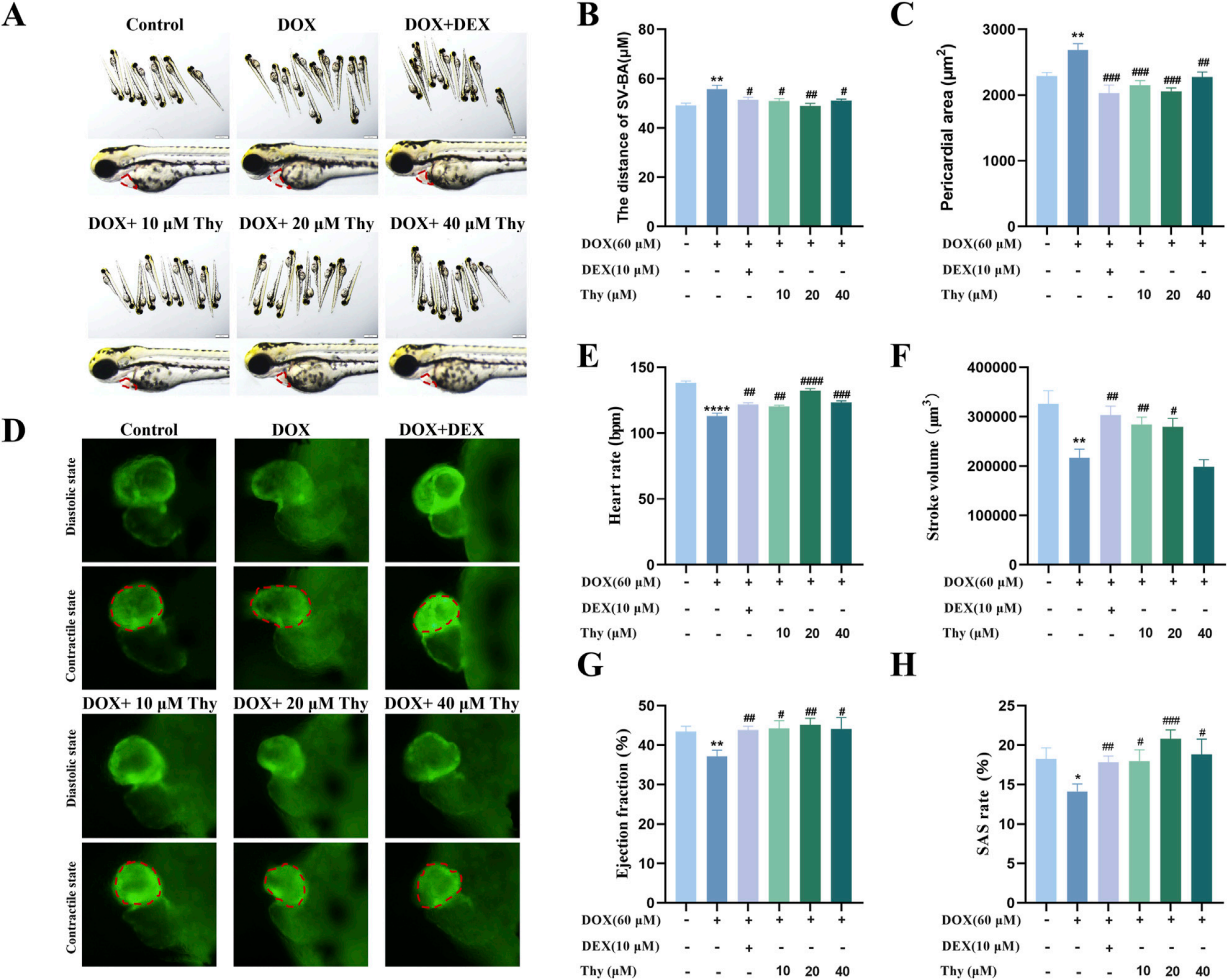
Effect of Thy on heart function in zebrafish with DOX-induced cardiomyopathy. **(A)** Morphological diagram of the zebrafish in each group, the red dotted box represents the heart area; **(B)** Statistical graph of the SV‒BA distance in zebrafish; **(C)** Statistical graph of the pericardial area in zebrafish; **(D)** Fluorescence in the zebrafish heart, the red dotted box represents the zebrafish ventricle; **(E)** Graph of the heart rate in zebrafish; **(F)** Graph of the stroke volume in zebrafish; **(G)** Graph of the ejection fraction in zebrafish; **(H)** Graph of the SAS rate in zebrafish. Compared with the blank group, ****p < 0.0001; Compared with the DOX model group, #p < 0.05, ##p < 0.01, ###p < 0.001, ####p < 0.0001.

At 24 h, compared with those in the blank control group, pericardial edema and the SV-BA distance were significantly greater in the DOX model group. Compared with the DOX model group, the DEX-positive control group and the Thy treatment group (60 μM DOX + 10, 20, 40 μM Thy) presented reduced pericardial edema (Figure 1A), decreased SV-BA (Figure 1B), and decreased pericardial area (Figure 1C). When the concentration of Thy was 20 μM, the reduction in DOX-induced pericardial edema and the increase in the SV-BA distance were the most significant.

### Effects of thy on heart function in zebrafish with DOX-induced cardiomyopathy

As shown in Figure 1D, after 24 h of drug administration, the ventricular volume in the DOX model group increased. Compared with those in the blank control group, the heart rate, stroke volume, ejection fraction, and SAS rate of the zebrafish in the DOX model group were significantly lower. Compared with the DOX model group, when the Thy concentration was 10 μM or 20 μM, the heart rate, stroke volume, ejection fraction, and SAS rate of the zebrafish significantly increased. With 40 μM Thy, the heart rate, ejection fraction and SAS rate of the zebrafish increased, but the stroke volume did not change (Figures 1E–H).

### Effects of thy on blood flow velocity in zebrafish with DOX-induced cardiomyopathy

**FIGURE 2:**
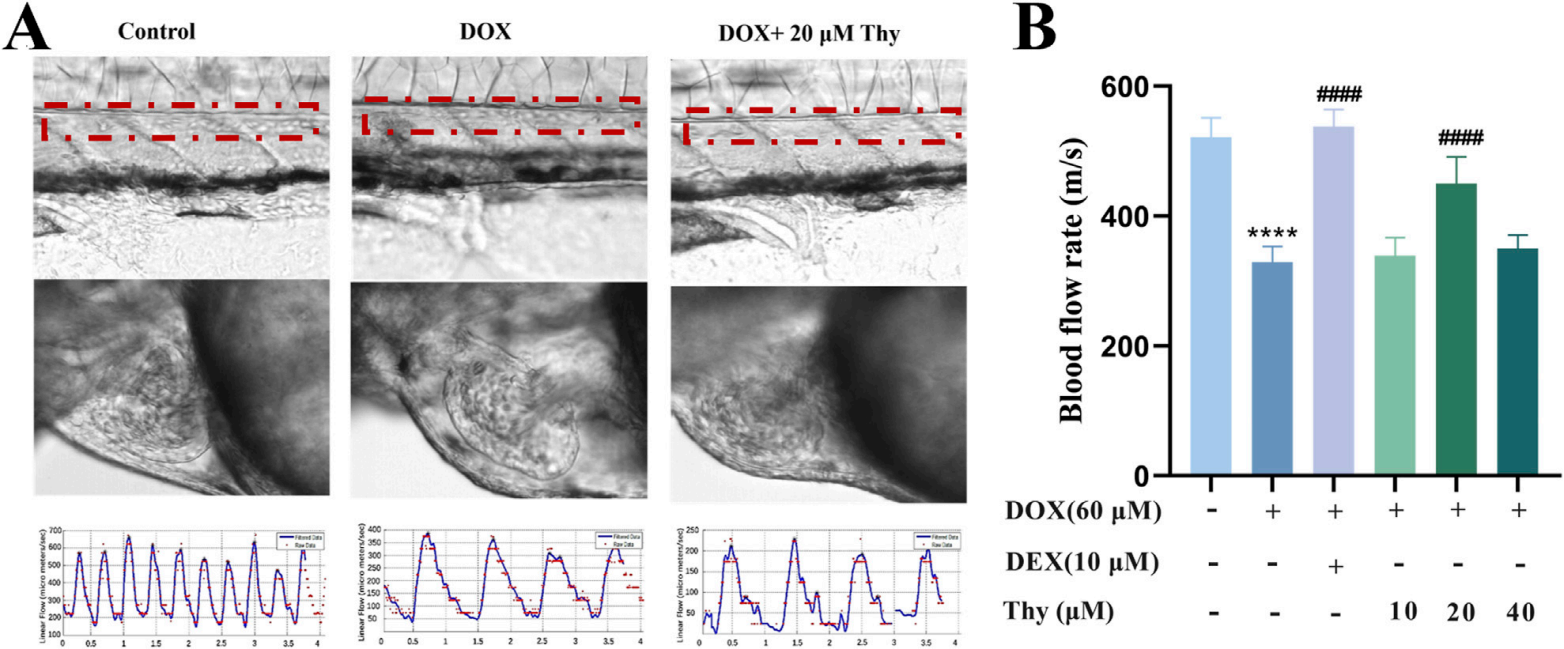
Effect of Thy on the blood flow rate in zebrafish with DOX-induced cardiomyopathy. **(A)** Schematic diagram of blood flow in the tail artery and heart of zebrafish; the red line indicates the artery; **(B)** Statistical graph of blood flow velocity in the tail artery of zebrafish. Compared with the blank group, ****p < 0.0001; Compared with the DOX model group, ####p < 0.0001.

As shown in Figures 2A,B, compared with that in the blank control group, the blood flow velocity in the tail of the zebrafish in the DOX model group was significantly lower. Compared with that in the DOX model group, the blood flow velocity in the DEX-positive control group and the 20 μM Thy treatment group was significantly greater and close to the level observed in the control group.

### Effects of thy on the erythrocyte area in the hearts of zebrafish with DOX-induced cardiomyopathy

**FIGURE 3:**
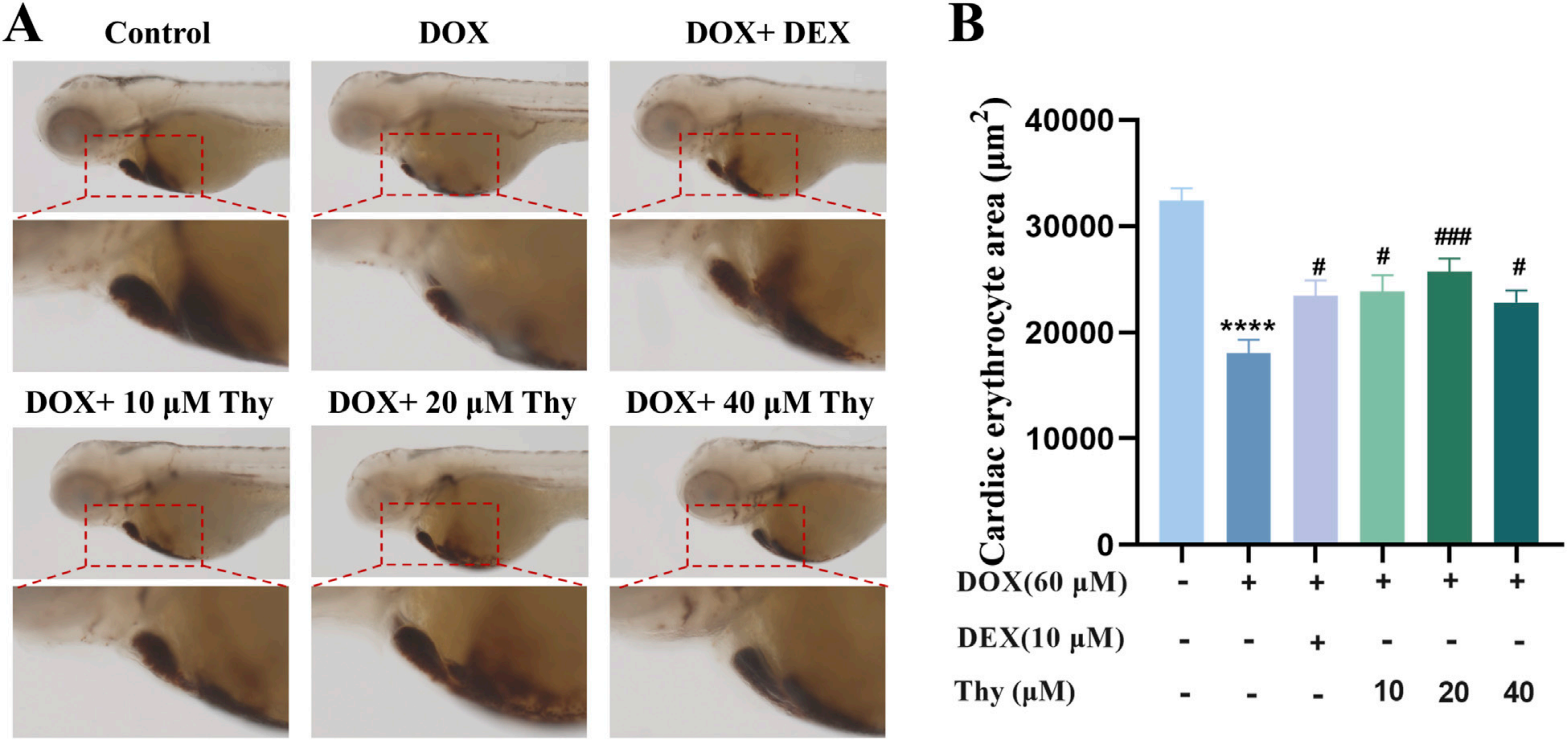
Effect of Thy on the erythrocyte area in the hearts of zebrafish with DOX-induced cardiomyopathy. **(A)** Adjacent O-dianisidine staining of the heart in zebrafish; **(B)** Statistical graph of the area of erythrocyte staining in the heart of zebrafish. Compared with the blank group, ***p < 0.001; Compared with the DOX model group, #p < 0.05, ##p < 0.01.

Twenty-four hours after drug administration, erythrocyte staining of the hearts of the zebrafish in each group was performed; the results are shown in Figure 3A. Compared with that in the blank control group, the area of erythrocyte staining in the DOX-treated zebrafish was significantly lower. Compared with that in the DOX model group, the area of staining area in the zebrafish in the DEX-positive control group and the Thy-treated group was significantly greater; 20 μM Thy had the best effect against DOX-induced myocardial ischemia (Figure 3B).

### Effects of thy on the apoptosis of cardiac cells in zebrafish with DOX-induced cardiomyopathy

**FIGURE 4:**
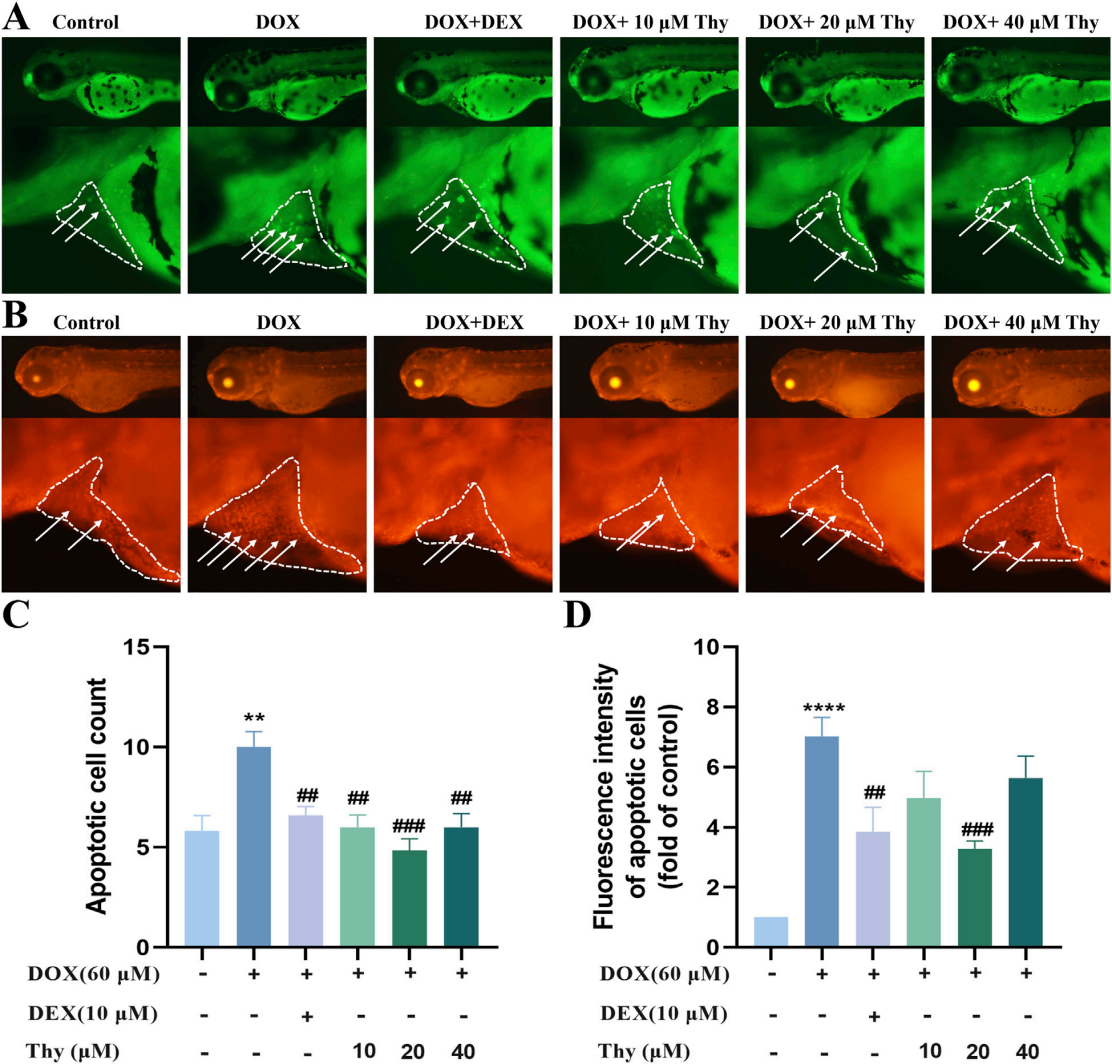
Effect of Thy on the apoptosis of cardiomyocytes in zebrafish with DOX-induced cardiomyopathy. **(A)** AO staining. The white arrowhead indicates cell apoptosis in the heart region, and the white line indicates pericardial edema; **(B)** TUNEL staining. The white arrowhead indicates cell apoptosis in the heart region, and the white line indicates pericardial edema; **(C)** Statistical graph of apoptotic cell numbers; **(D)** Statistical graph of fluorescence intensity of apoptotic cells. Compared with the blank group, **p < 0.01, ****p < 0.0001; Compared with the DOX model group, ##p < 0.01, ###p < 0.001.

The AO staining and TUNEL staining results are shown in Figures 4A,B. Compared with those in the hearts of zebrafish in the blank group, there were more fluorescent-labeled apoptotic cells in the hearts of zebrafish in the DOX model group. Compared with those in the DOX model group, the number of fluorescent dots and fluorescence intensity in the heart area was significantly decreased after treatment with DEX and 10, 20 or 40 μM Thy, and the antiapoptotic effect on cardiac cells was most significant when the Thy concentration was 20 μM (Figures 4C,D).

### Effects of thy on the morphology of heart tissue in zebrafish with DOX-induced cardiomyopathy

**FIGURE 5:**
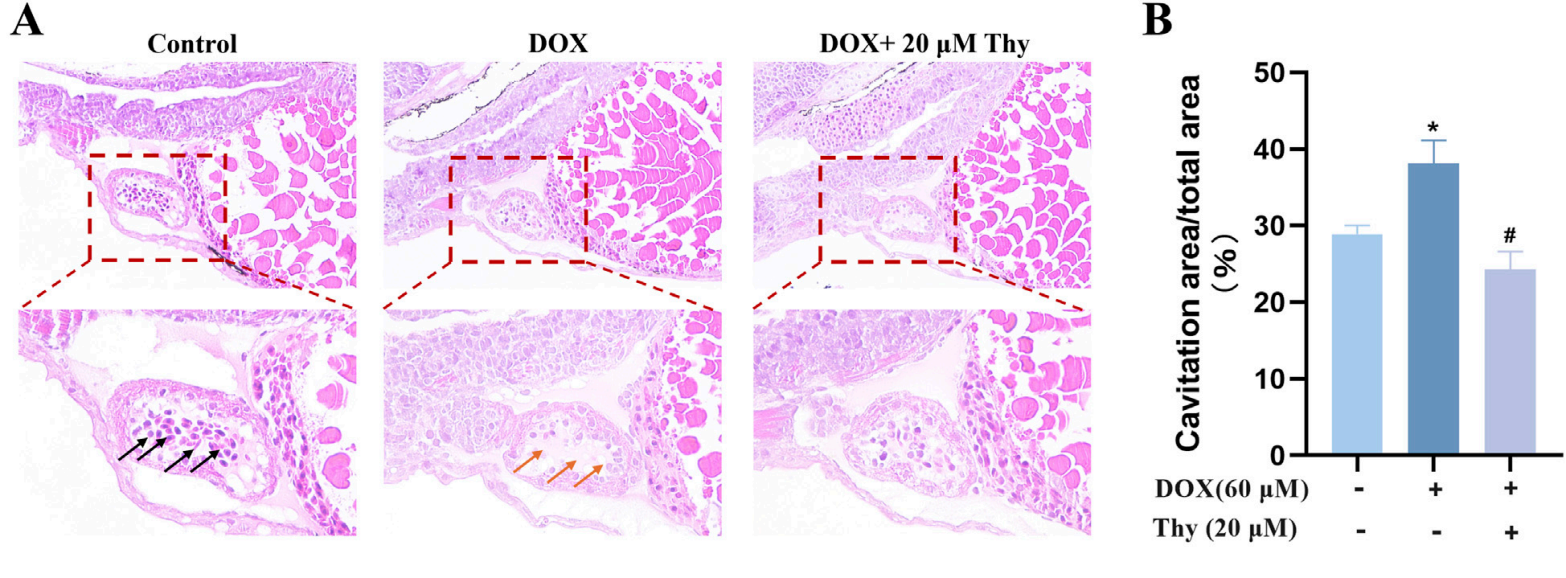
Effect of Thy on the morphology of heart tissue in zebrafish with DOX-induced cardiomyopathy. **(A)** Black arrows indicate normal intercellular gaps and tightly arranged myocardial cells; Orange arrows indicate large intercellular gaps and disordered cardiomyocytes. **(B)** Statistical graph of heart vacuolation in zebrafish. Compared with the blank group, *p < 0.05; Compared with the DOX model group, #p < 0.05.

The H&E staining results are shown in Figure 5A. The sizes of the atria and ventricles in zebrafish in the normal control group were normal, and the intercellular gap between cardiomyocytes was normal; cardiomyocytes were tightly arranged (black arrow). In the DOX model group, the atria and ventricles in zebrafish were significantly dilated and enlarged, and the intercellular gap between cardiomyocytes was larger than that observed in the normal control group; cardiomyocytes were disorderedly arranged (indicated by the orange arrow). Compared with those in the DOX model group, the degree of dilatation of the heart in zebrafish was significantly greater after treatment with 20 μM Thy, and cardiomyocytes were arranged more tightly. The degree of vacuolization in the heart tissue is shown in Figure 5B. Compared with the control group of zebrafish, the vacuolization area in the DOX model group was significantly larger. Compared with the DOX model group, the vacuolization area in the zebrafish heart decreased after treatment with 20 μM Thy.

### Effects of thy on lipid accumulation in the hearts of zebrafish with DOX-induced cardiomyopathy

**FIGURE 6:**
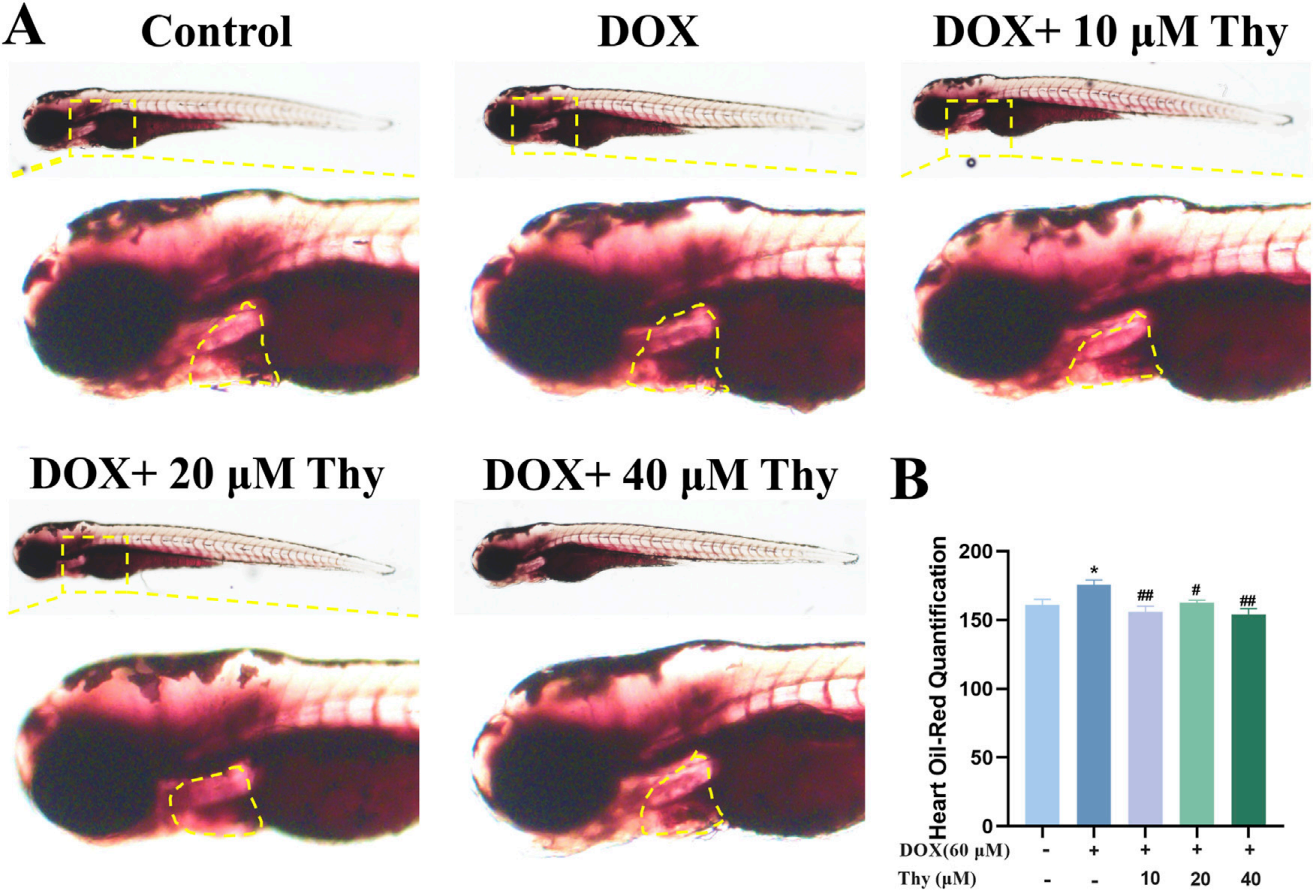
Effect of Thy on lipids in the hearts of zebrafish with DOX-induced cardiomyopathy. **(A)** Oil red O-stained zebrafish; the yellow dotted line indicates the heart region; **(B)** Statistical graph of the intensity of the Oil red O staining in zebrafish. Compared with the blank group, *p < 0.05; Compared with the DOX model group, #p < 0.05, ##p < 0.01.

After drug administration, Oil red O staining of the zebrafish in each group was performed to observe the effect of Thy on lipid accumulation in the hearts of zebrafish with DOX-induced cardiomyopathy. As shown in Figure 6, compared with those in zebrafish in the blank control group, the hearts of zebrafish in the DOX model group were darker, as shown by Oil red O staining. Compared with that in the DOX model group, the degree of staining in the hearts of zebrafish in the 10, 20, and 40 μM Thy treatment group was significantly lower.

### RT‒qPCR validation

**FIGURE 7:**
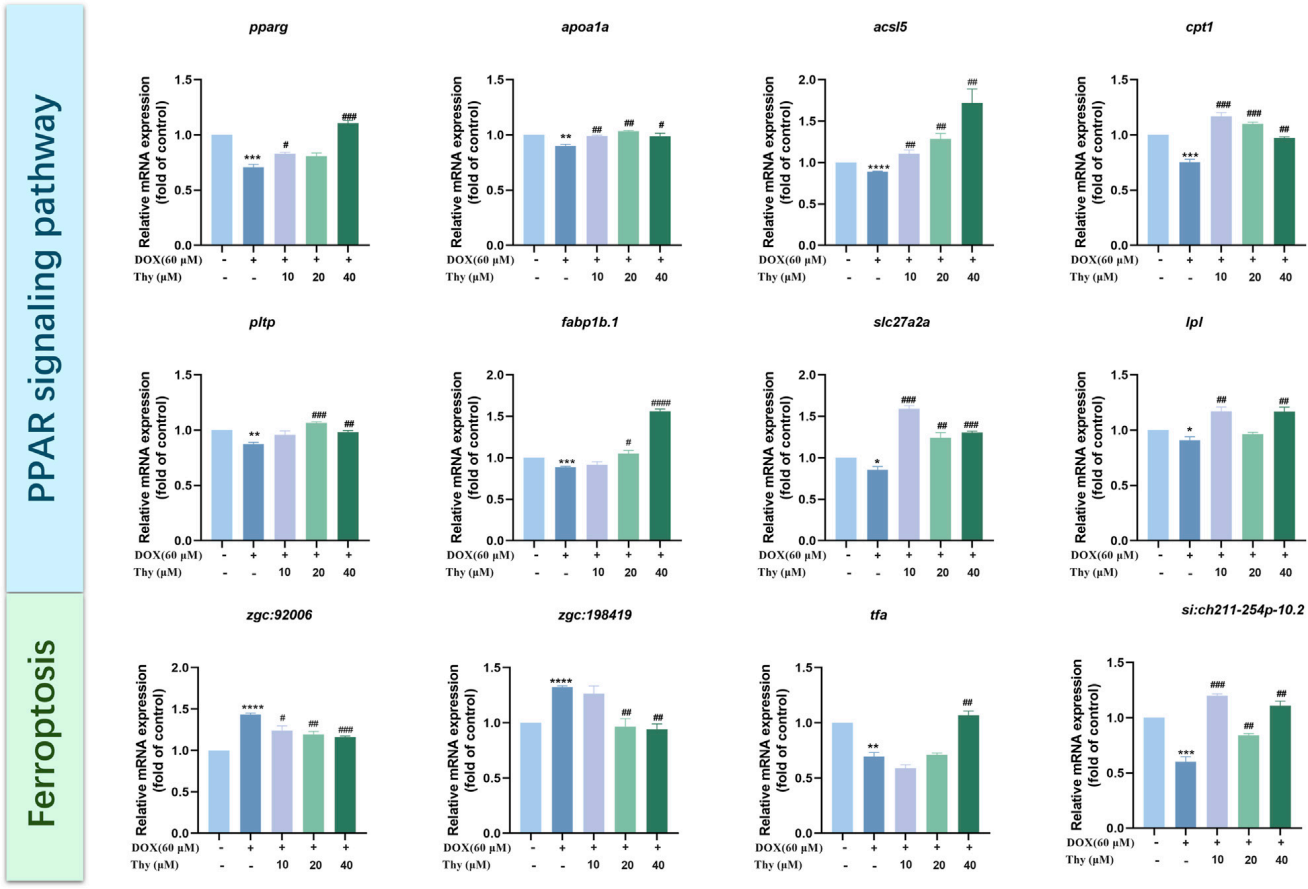
Effect of Thy on gene expression in zebrafish with DOX-induced cardiomyopathy. Compared with the blank group, *p < 0.05, **p < 0.01, ***p < 0.001, ****p < 0.0001; Compared with the DOX model group, #p < 0.05, ##p < 0.01, ###p < 0.001, ####p < 0.0001.

The mRNA expression levels of genes related to the PPAR signaling pathway were assessed via RT‒qPCR. Compared with that in the blank group, the mRNA expression levels of *pparg, apoa1a, acsl5, cpt1, pltp, fabp1b.1, slc27a2a, and lpl* were lower. Compared with those in the DOX model group, the mRNA expression levels of *pparg, apoa1a, acsl5, cpt1, slc27a2a, and lpl* were higher in the 10 μM Thy treatment group; in the 20 μM Thy treatment group, the mRNA expression levels of *apoa1a, acsl5, cpt1, pltp, fabp1b.1, and slc27a2a* were higher, and the expression of the other genes presented no significant changes; and in the 40 μM Thy treatment group, the mRNA expression levels of *pparg, apoa1a, acsl5, cpt1, pltp, fabp1b.1, slc27a2a, and lpl* were higher (Figure 7).

The mRNA expression levels of ferroptosis pathway-related genes were assessed via RT‒qPCR. Compared with those in the blank control group, the expression levels of *zgc:198419* and *zgc:92006* tended to be higher and the mRNA expression levels of *tfa* and *si:ch211-254p-10.2* were lower in the DOX model group. Compared with those in the DOX model group, the mRNA expression levels of *si:ch211-254p-10.2* were higher and the mRNA expression levels of *zgc:92006* were lower in the 10 μM Thy treatment group; in the 20 μM Thy treatment groups, the mRNA expression levels of *zgc:198419* and *zgc:92006* were lower, and the mRNA expression levels of *si:ch211-254p-10.2* were higher; and in the 40 μM Thy treatment group, the mRNA expression levels of *zgc:198419* and *zgc:92006* were lower, and the mRNA expression levels of *tfa* and *si:ch211-254p-10.2* were higher (Figure 7).

## Discussion

In this study, *Tg (cmlc2: EGFP)* and wild-type AB zebrafish were used as research objects, and the morphology and function of the hearts of the zebrafish were observed via fluorescence microscopy. A DOX-induced cardiomyopathy model was established with 60 μM DOX, and model recapitulated the symptoms of DCM. Compared with the blank group, the DOX model group presented significant pericardial edema; a higher SV-BA; a significantly lower heart rate, stroke volume, SAS rate, and ejection fraction; slower blood flow; higher number of apoptotic cells; lower number of erythrocytes in the heart region; and cardiomyocyte disruption. Compared with that in zebrafish in the DOX group, cardiotoxicity was alleviated in the Thy treatment group, a finding that was consistent with the effects of the positive control drug. The results showed that Thy had anti-DOX-induced cardiomyopathy activity.

PPAR is widely expressed in myocardial cells and is involved in the regulation of energy metabolism, proliferation, differentiation, development and death of myocardial cells. PPARs can also regulate lipid homeostasis. The aggregation of lipid peroxides or an imbalance in the antioxidant system can cause ferroptosis (Chen et al., 2021). Some studies have shown that DOX can cause excessive lipid accumulation by affecting the PPAR signaling pathway, thereby inducing cardiomyopathy (Wagner and Wagner, 2020a; Wagner and Wagner, 2020b). The results of this study suggest that the PPAR signaling pathway and the ferroptosis pathway play important roles in the development DOX-induced cardiomyopathy in zebrafish. Therefore, we focused on the regulatory effect of Thy on the PPAR signaling pathway and the ferroptosis pathway.

We used RT‒qPCR to analyze the expression of genes related to the PPAR signaling pathway and ferroptosis pathway to elucidate the mechanism by which Thy alleviates DOX-induced heart injury. There are three subtypes of PPARs: *pparα, pparβ, and pparg. Pparg* is a ligand-activated transcription factor and regulates the expression of multiple genes and the transcription of genes related to lipid metabolism in different ways, including binding to fatty acids and their derivatives (Wang et al., 2024; Cai et al., 2019). Hannah E. Wilson et al. reported that the *pparg* agonist rosiglitazone decreased lipid accumulation (Wilson et al., 2021). Carnitine acyltransferase 1 (Cpt1) plays an important role in lipid metabolism, and the upregulation of *cpt1b* expression is closely related to lipid β-oxidation (Zhang et al., 2019; Ngo et al., 2023). Apolipoprotein A1 (Apoa1) is the major apolipoprotein of plasma high-density lipoprotein (HDL) is involved in the regulation of intracellular lipid levels (Cochran et al., 2021). One study revealed that decreased *apoa1* expression promoted the generation of reactive oxygen species (ROS), leading to inflammation and oxidative stress (Maarfi et al., 2023). The Acsl5 protein is a key enzyme in the activation of fatty acids and plays a critical role in lipid metabolism (Qin et al., 2023). Moreover, phospholipids and other related molecules are transported between lipoproteins and cell membranes, thus playing a key role in cell signal transduction and metabolic regulation and affecting ferroptosis sensitivity (Gnanapradeepan et al., 2022). The main function of fatty acid binding protein 1 (Fabp1) is transporting lipophilic substrates and long-chain fatty acids in cells. Studies have shown that *Fabp1* can reduce drug-induced oxidative stress, reduce cytotoxicity, and exert a protective effect (Mukai et al., 2017). Lipid peroxidation can lead to ferroptosis, and *fabp1* can regulate the level of intracellular oxidative stress, thereby affecting the development of intracellular ferroptosis (Jian et al., 2023). The RT‒qPCR results in this study suggested that DOX led to the abnormal expression of genes related to the PPAR signaling pathway (*pparg, cpt1, apoa1a, acsl5, pltp, fabp1b.1, slc27a2a,* and *lpl*) and that Thy significantly reversed the abnormal expression of these genes. The Oil Red O staining results revealed lipid accumulation in the heart area of the zebrafish in the DOX model group; however, after treatment with 20 μM Thy, the intensity of Oil Red O staining in the heart area of the zebrafish significantly decreased. Therefore, the results of this study suggest that Thy decreases lipid accumulation in the heart of zebrafish and reduces ferroptosis by activating the PPAR signaling pathway, thereby exerting anti-DOX-induced heart injury effects.

Ferroptosis is an iron-dependent form of programmed cell death that is caused by the excessive accumulation of lipid peroxides on the cell membrane. Some studies have shown that DOX-induced cardiomyopathy is closely related to the ferroptosis pathway. The ferroptosis inhibitor ferrostatin-1 (Fer-1) has been shown to significantly reduce the mortality of mice with DOX-induced cardiomyopathy (Kitakata et al., 2022). In this study, Thy significantly reversed the abnormal expression of ferroptosis pathway-related genes caused by DOX, for example, *zgc:198419, zgc:92006, tfa*, and *si:ch211-254p-10.2*. These findings suggest that Thy alleviates DOX-induced heart injury by inhibiting ferroptosis.

In summary, this study revealed that Thy has a significant effect on DOX-induced cardiomyopathy. The mechanism of action of Thy may involve the activation of the PPAR signaling pathway to reduce lipid accumulation in the heart of zebrafish, thereby alleviating ferroptosis. This study provides a theoretical basis and an experimental basis for the development of novel drugs against DOX-induced cardiomyopathy.

## Data Availability

The raw data supporting the conclusion of this article will be made available by the authors, without undue reservation.
