# Supplementary material for: Thymidine exerts anti-doxorubicin-induced cardiomyopathy effect through the regulation of the PPAR signaling pathways and ferroptosis pathways
Source: Front Pharmacol. 2025 Sep 30;16:1524167. doi: 10.3389/fphar.2025.1524167 (PMC12517584; doi:10.3389/fphar.2025.1524167)
Supplement: Supplementary file 1 [file Table1.doc]

Supplementary Table 1. Sequence of Primers Used in qPCR

| Genes | Sequence | Primer orientation |
| --- | --- | --- |
| *β-actin* | 5′- GAGCTACGAGCTGCCTGACG -3′ | Forward |
|  | 5′- CCTAGAAGCATTTGAGGTGG -3′ | Reverse |
| *pparg* | 5′-CAGTTTGCAGAGAACAGCGT-3′ | Forward |
|  | 5′-GGCTCTTCTTGTGTATGCGG-3′ | Reverse |
| *apoa1a* | 5′-TAAGCTGACCGAGCGTCTTG -3′ | Forward |
|  | 5′-TCTGTGCGAATGTGGTCCTC -3′ | Reverse |
| *acsl5* | 5′-AGCCATGCTGACCCATGGAC -3′ | Forward |
|  | 5′-ATGGACACGTCGGAGTGCCC -3′ | Reverse |
| *cpt1ab* | 5′-TGCAGGGGAGATGTAGACCA -3′ | Forward |
|  | 5′-TGACAGTCCACTTCATCGGC -3′ | Reverse |
| *pltp* | 5′-CAATGAAGGGCAAACGCCTC-3′ | Forward |
|  | 5′-CACCCCTCGCTTGGTGTAAT-3′ | Reverse |
| *fabp1b.1* | 5′-GAGCAGGGCGTCATCACTAT -3′ | Forward |
|  | 5′-AGTTTACCATCCGCAAGGCT -3′ | Reverse |
| *slc27a2a* | 5′-CGTGCTTCTCCACACTCGAT -3′ | Forward |
|  | 5′-TGCATCCCGGTAAGTGTAGC -3′ | Reverse |
| *lpl* | 5′-ACGGGTGTTGGTGTGAAGAA -3′ | Forward |
|  | 5′-CCCACTTTTGCACATGGACG -3′ | Reverse |
| *zgc:92066* | 5′-GGCTTTCTACTTTGACCGGGA -3′ | Forward |
|  | 5′-ATCGCGCTCATTCCCCGTC -3′ | Reverse |
| *zgc:198419* | 5′-AACGGGACGATGTAGCCCTTAAT -3′ | Forward |
|  | 5′-ACAATGCGTCCACCTCTCTTG -3′ | Reverse |
| *tfa* | 5′-TGGGGCTTTCAGGTGTCTTG -3′ | Forward |
|  | 5′-TCCTTTGCCCAGTCCTTTCC -3′ | Reverse |
| *si:ch211-254p10.2* | 5′-AAATCTGGGATGGGTGGCTG -3′ | Forward |
|  | 5′-TCAATCCGCCTCATCGTAGG -3′ | Reverse |
